# An Integrative Review on the Feasibility and Acceptability of Delivering an Online Training and Mentoring Module to Volunteers Working in Community Organizations

**DOI:** 10.3389/fdgth.2021.688982

**Published:** 2021-10-14

**Authors:** Taylor G. Hill, Jodi E. Langley, Emily K. Kervin, Barbara Pesut, Wendy Duggleby, Grace Warner

**Affiliations:** ^1^Department of Psychology and Neuroscience, Dalhousie University, Halifax, NS, Canada; ^2^Faculty of Health, Dalhousie University, Halifax, NS, Canada; ^3^Faculty of Medicine, Dalhousie University, Halifax, NS, Canada; ^4^Nursing, University of British Columbia, Okanagan, BC, Canada; ^5^Nursing, University of Alberta, Edmonton, AB, Canada; ^6^School of Occupational Therapy, Dalhousie University, Halifax, NS, Canada

**Keywords:** volunteers, online training, older adults, community support, program implementation

## Abstract

**Background:** Volunteer programs that support older persons can assist them in accessing healthcare in an efficient and effective manner. Community-based initiatives that train volunteers to support patients with advancing illness is an important advance for public health. As part of implementing an effective community-based volunteer-based program, volunteers need to be sufficiently trained. Online training could be an effective and safe way to provide education for volunteers in both initial training and/or continuing education throughout their involvement as a volunteer.

**Method:** We conducted an integrative review that synthesized literature on online training programs for volunteers who support older adults. The review included both a search of existing research literature in six databases, and an online search of online training programs currently being delivered in Canada. The purpose of this review was to examine the feasibility and acceptability of community-based organizations adopting an online training format for their volunteers.

**Results:** The database search identified 13,626 records, these went through abstract and full text screen resulting in a final 15 records. This was supplemented by 2 records identified from hand searching the references, for a total of 17 articles. In addition to identifying Volunteers Roles and Responsibilities; Elements of Training; and Evaluation of Feasibility and Acceptability; a thematic analysis of the 17 records identified the categories: (1) Feasibility Promoting Factors; (2) Barriers to Feasibility; (3) Acceptability Promoting Factors; and (4) Barriers to Acceptability. Six programs were also identified in the online search of online training programs. These programs informed our understanding of delivery of existing online volunteer training programs.

**Discussion:** Findings suggested that feasibility and acceptability of online training were promoted by (a) topic relevant training for volunteers; (b) high engagement of volunteers to prevent attrition; (c) mentorship or leadership component. Challenges to online training included a high workload; time elapsed between training and its application; and client attitude toward volunteers. Future research on online volunteer training should consider how online delivery can be most effectively paced to support volunteers in completing training and the technical skills needed to complete the training and whether teaching these skills can be integrated into programs.

## Statement

Our review synthesized evidence on web-based training programs for volunteers who deliver services to older adults. This practical research topic is a public health issue, with sparse literature linking knowledge at the volunteer, training program, and older adult levels. While there is abundant knowledge of the broader topics of volunteerism, our review of online training provides timely knowledge relevant to the current pandemic circumstances (and the post-pandemic reality). In addition to the unique structure of the research topic, this evidence synthesis provides a practical foundation for transitioning volunteer training online, or for strengthening existing online training programs. Knowledge garnered from this synthesis could help community-based organizations in program delivery and design consider how online delivery can be most effectively placed to increase volunteer access to training.

## Introduction

Population projections suggest that globally the number of individuals 65 years and over will more than double by 2050 (1). Accompanying global population aging is a rise in chronic and degenerative diseases. These demographic changes are met with increased demand for healthcare supports and services (1). Volunteer programs that offer services to older persons can assist them in accessing healthcare in an efficient and effective manner. Multiple studies have shown positive patient outcomes of volunteers providing in-home services for dementia care [e.g., (2)], support with mobilization in nursing homes (3) and educational health-promoting sessions in the community (4–7).

Older persons are often appropriate recipients of the public health approach to palliative care, that takes an upstream community-based approach to palliative care, by identifying and supporting individuals early in their trajectory toward end of life with the goal of enhancing their quality of life, symptom management, and mental health (8–10). The public health approach to palliative care entails the integration of a broad group of stakeholders who can be mobilized to support community-living individuals as they deal with serious illnesses (11, 12). Community-based initiatives that train volunteers to support patients with advancing illness is an important component to implementing the public health approach to palliative care (3, 13). As part of implementing an effective community-based volunteer-based program volunteers need to be sufficiently trained.

Although relatively new, online volunteer training has emerged as an effective and feasible way to educate a large group of volunteers in a timely manner (14, 15). As opposed to in-person training, online training addresses the barriers of time, accessibility, and cost. Online training could provide a more accessible learning environment without compromising the integrity of the training. Frendo (15) analyzed three different modes of delivery for volunteer training (online vs. face-to- face vs. blended) and found that although face-to-face training was highly regarded in creating connections with other volunteers, online training met volunteers' motivational needs when opportunities were provided for learners to interact in real-time with people from a distance. The benefits to online volunteer training may outweigh the barriers. Although technical difficulties and limited interaction amongst participants can create barriers during training, this is offset by tangible benefits such as the ability to draw participants from a larger geographic region, provide flexible training times, and engage participants in cross cultural learning (14, 15). Given these benefits understanding how to provide acceptable and feasible online training is an important initial step.

Acceptability and feasibility are key considerations when implementing innovative programs, such as educational programs (16). While there is a notable absence of concrete definitions for acceptability in the context of online volunteer training (16), an innovation is often considered acceptable when its benefits are identifiable and/or it addresses previous systemic, social, or educational challenges (17). The intended users' reaction to the innovation is perhaps the ultimate test of acceptability. This is often assessed by indicators of satisfaction, intent to continue use, perceived appropriateness, and fit with the culture (18). Ensuring acceptability to recipients of a program and to those who deliver it is essential. It can impact whether the program is delivered as intended, and by extension whether there are positive outcomes for recipients (19, 20). Furthermore, gauging feasibility before launching a new program is essential for identifying and planning to address logistical challenges (17). Perhaps most importantly, assessing feasibility can help ensure the program is compatible with the available resources of those who intend to implement the training (21–25). Feasibility is an important aspect to measure given the limited resources most community- based organizations have available.

Sustainability is also a potential advantage of online training programs. Beyond online training providing a format that increases training capacity, it could also facilitate continuation of a community-based program in which education is an important component. If the training is acceptable and feasible to implement, organizations that transition in person training to online could realize benefits in terms of cost effectiveness and training quality. Online training could be an effective and safe way to provide education for volunteers in both initial training and/or continuing education throughout their involvement as a volunteer.

### Aims

The aim of this review was to examine the feasibility and acceptability of community-based organizations adopting an online training format for their volunteers by synthesizing evidence from the research and gray literature on online training/coaching/mentoring programs for volunteers delivering services to older persons. This review intends to examine factors that facilitate, or hinder, an organization successfully implementing online training. These factors will provide a basis for recommendations for adopting online training and a foundation for future research. For instance, identifying barriers to feasible and acceptable online training programs for volunteers can help organizations identify potential issues when designing programs so they can address them early in the development phase. Likewise, understanding factors that facilitate feasibility and acceptability can help them maximize factors that can lead to successful adoption. Ultimately, we hope this review will inform future online volunteer training efforts to support older adults in the community.

**The research question directing this integrative review is:**

How feasible and acceptable is it for community-based organizations supporting older adults to adopt an online training format for their volunteers?

**Two sub-questions are:**

What are the barriers to feasible and acceptable online training for volunteers who support older adults in the community?

What are the facilitators of feasible and acceptable online training for volunteers who support older adults in the community?

## Methods

To answer the research question, an integrated review was conducted to synthesize evidence from the research and gray literature on online training/coaching/mentoring programs for volunteers who deliver services to older persons. An integrative review is an umbrella term used to describe a synthesis method for integrating qualitative and quantitative data, such as mixed studies reviews and critical interpretive synthesis (26). These types of reviews provide a comprehensive overview of a subject area to inform a specific problem.

This review will aid in understanding how the online training programs were conducted and the perspectives of training participants and organizational administrators on acceptability, feasibility, and effectiveness. Two methods were used for the review: (A) a database search of the existing research literature, and (B) an online search of existing online volunteer training programs currently being delivered in Canada. The methods are described for the database searches and online search strategy separately. The database search of existing research literature was broadly set to training for volunteers supporting adults. The intent of the online search of existing volunteer training programs was to identify programs that offer training for volunteers supporting older adults.

### Method A: Database Search of the Existing Literature

#### Search Strategy

**Figure 1:**
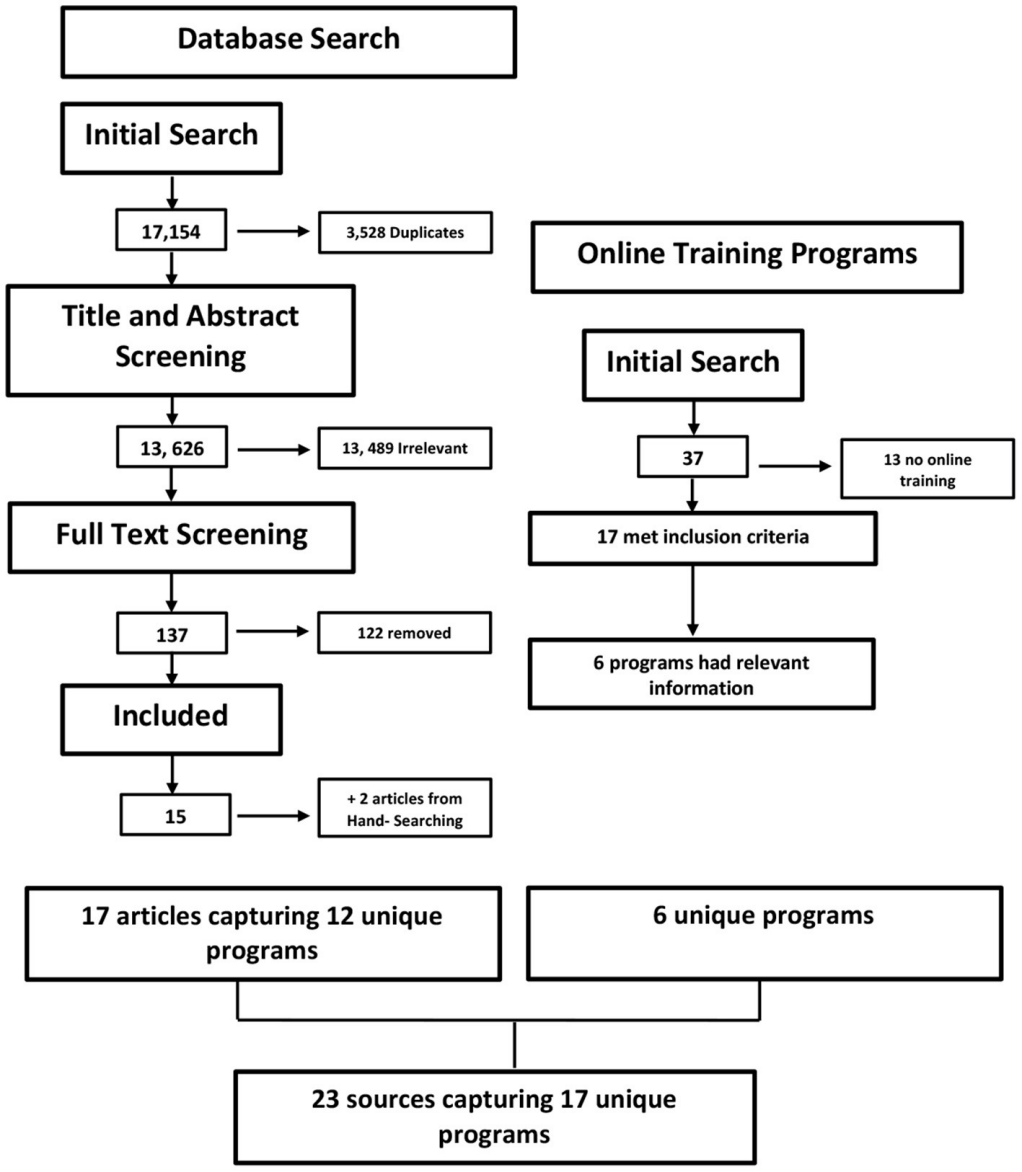
PRISMA Review flow diagram for all search strategies broken down to the two separate search strategies, (1) Database Search and (2) Online Training Programs.

In collaboration with a librarian a search strategy was developed using the key concepts of volunteer training, navigation, mentoring, coaching, online, virtual. The database search was conducted on MEDLINE (ovid), CINAHL (EBSCO), EMBASE (Elsevier), PsycInfo (EBSCO), Embase (Elsevier), Social work abstracts (EBSCO), ERIC (ProQuest). The search was completed in June 2020. Search results are in Figure 1.

To be included, studies: (a) written in English, (b) focused on adults being trained to provide support to adults in an unpaid setting, (c) part of an intervention that utilized online training modules or coaching/mentoring programs, (d) either theoretical (e.g., best practices for designing and delivering training), empirical (e.g., protocol, evaluation), gray literature (e.g., online organizations providing training), or reviews, and (e) described outcomes such as perspectives of training participants, feasibility, acceptability, or effectiveness of training. Studies were excluded if they had student, university, school, pedagogy, classroom, or undergraduate in the title.

Once the search was completed records were downloaded into a reference management (Mendeley) software to identify duplicate records, then transferred into Covidence, an online tool used for organizing and expediting systematic reviews (27). Three reviewers scanned the same 50 articles to validate the inclusion process then met to review their results. The fourth reviewer was added later and trained independently by one of the original reviewers. There were two stages to the review process. The title and abstract phase focused on identifying articles that reflected key study concepts and the full text stage explored whether key concepts are operationalized in a way that could address the research question.

The team of four research members independently scanned the title, abstract, or both sections of every record retrieved, with at least two reviewers per article. Potentially relevant papers were retrieved in full and their citation details imported into the Covidence software. Next, two independent reviewers assessed the full text of selected articles in detail against the inclusion criteria. When there were disagreements on inclusion/ exclusion, the two reviewers discussed the articles to determine whether the article should be included. In addition, reference lists in the included articles were reviewed to identify additional relevant literature.

A 2020 PRISMA flow chart was created as a visual summary of the number of records identified through database searching and other sources, as well as the number of records included and excluded (http://www.prisma-statement.org). Data was extracted using Covidence and pre-coded data extraction categories, see Appendix for detailed template.

### Method B: Online Search of Existing Online Training Programs

#### Search Strategy

Canadian websites were scanned to identify and extract data on online volunteer training programs specific to older adults. Two separate search methods were used for the initial online program search. For the primary search a Google search was conducted to identify organizations focused on end-of-life care in Canada using the quality of end-of-life coalition as a beginning point. This search was able to identify provincial and territorial hospice societies. The following steps were completed:

1. Starting with the End-of-life coalition (https://www.chpca.ca/projects/the-quality-end-of-life-care-coalition-of-canada/) all organizations listed on the website were explored.
2. Each provincial and territorial hospice associations' websites were explored to identify specific lists of hospices within each area.
3. The website: http://www.canadian-universities.net/Volunteer/Seniors.html was investigated to volunteer opportunities by “Seniors.” All suggested organizations were investigated, those without a website and were excluded.
4. All the links in each province listed on the following website: https://volunteer.ca/index.php?MenuItemID=422 were identified and each website was filtered by the option indicating virtual or by distance.

In the second Google search a search of organizations focused on older adults was added, the search strategy utilized a combination of key words, this was followed by removing one word/phrase at a time. The key words were: “older persons” OR seniors OR elderly; Hospice OR palliative OR “end of life”; Volunteer; Training OR online OR resources OR education; Community OR care OR support; and Canada. The first 10 Google pages were searched for each possible word combination to identify and scan potential websites to make sure it was specific to older persons. Then, the website was explored to identify whether there was volunteer training and if it was online. This search resulted in 10 possible programs, two of these programs had already been identified in the primary search, eight possible programs were added to the database.

Furthermore, volunteer.ca, a Pan-Canadian Volunteer Matching Platform was searched using the criteria of any location in Canada, volunteering as an individual on an ongoing basis, and serving seniors. Separate searches were conducted for each age group of volunteers (i.e., 19–25, 26–35, 36–55, 56–70, 71+); no new programs were identified.

When appropriate websites were located the following data were extracted: name of organization; mission/aim/focus of organization (i.e., provide meals on wheels); scope of organization; volunteer characteristics and responsibilities; and description of the volunteer training program (e.g., name, length/frequency; materials, content, additions to training); evaluations. The extracted data was organized using Excel.

### Data Analysis

Evidence from the research and gray literature on online training/coaching/mentoring programs for volunteers who deliver services to older persons was synthesized. Data analysis was completed in five phases: data reduction, data display, data comparison, conclusion drawing, and verification (26). For data reduction, a classification system was developed to cluster similar categories of data, this data was entered into a table (Appendix) to display the results of data reduction and facilitate data comparison. An approach similar to thematic analysis was used to identify commonalities, contrasts, and other relationships within the data table through an iterative process (28). The results of the data comparison and verification phases is both a narrative of the findings and a display of the data using tables to expand interpretations. Information on online programs for training volunteers was assessed for the presence of the barriers and facilitators found in the literature.

In order to effectively assess the level of evidence we chose an appraisal classification system that had separate classifications for both qualitative and quantitative studies and did not privilege quantitative over qualitative studies, Tomlin and Borgetto's (29) research pyramid. This pyramid breaks down articles into 4 separate categories (descriptive, experimental, outcomes, and qualitative research) to evaluate the level of evidence of studies included from different study designs. Each category describes four levels of evidence that go from the most to least rigorous. Descriptive research is divided into (1) Systematic reviews of related descriptive studies, (2) Association, correlational studies, (3) Multiple- case studies (series), normative studies, descriptive surveys, (4) Individual case studies. Experimental research is divided into (1) Meta- analyses of related experimental studies, (2) Individual (blinded) randomized controlled trials, (3) Controlled clinical trials, (4) Single- subject studies, Outcomes research is divided into (1) Meta- analyses of related outcomes studies, (2) Pre-existing groups comparisons with covariate analysis, (3) Case- control studies; pre-existing groups comparisons, (4) One-group pre- post studies. Finally qualitative research is divided into (1) Meta- synthesis of related qualitative studies, (2) Group qualitative studies with more rigor (a, b, c), (3) Group qualitative studies with less rigor- (a) Prolonged engagement with participants, (b) Triangulation of data (multiple sources), (c) Confirmation of data analysis and interpretation (peer and member checking), (4) Qualitative studies with a single informant.

## Results

The database search of the existing research literature, and online search of existing online training programs identified 23 sources that provided data on 16 unique programs; two programs are reported in 4 separate sources (Health TAPESTRY & Project HEAL).

### Method A: Database Search

A database search of the existing literature identified 17 articles. The programs involved working with anyone from seniors, to cancer survivors and individuals struggling with fertility. Although using different pedagogical theories, and e-learning strategies, self-paced learning modules were used nine times whereas a mixture of self-paced and live was used twice. Programs were open to any volunteers with three having restrictions to be at least 18 years of age, and six having restrictions to demographic characteristics; namely, race (4), sex (2), sexuality (1). There were also programs that limited volunteers to those with prior experience (1) or previous personal experience with disease (4). Training topics ranged across all programs, seven covered communication tips, nine covered science behind disease, five discussed ethics and four discussed the role of the volunteer and four programs discussed the use of technology in their training. Article information reflected in Table 1 was used to assess the level or quality of evidence (29).

**Table 1:** Characteristics of volunteer training programs from the database search.

**Program name**	**References**	**Level of evidence (Tomlin)**	**Years delivered**	**Purpose of study and/or program**	**Volunteer role**	**Inclusion criteria for volunteers**
Health TAPESTRY	Oliver et al. (30)	Descriptive Research: Level 4: Individual Case Study	Not reported	The Health TAPESTRY approach is a model of care which aims to overcome some of these barriers in the health care system and truly shift care from reactive to proactive care. This program had volunteers working with older adults.	Support Person	18 years or older, vulnerable sector check, TB skin test
	Dolovich et al. (31)	Outcome Research: Level 4: One group pre-post studies Qualitative Research: Level 2: Group Qualitative Studies with more rigor- prolonged engagement with participants and triangulation of data (multiple sources)	January to December 2016	To (a) explore the effectiveness of the training on volunteer knowledge, skills, and self-efficacy; (b) understand the feasibility of implementing the volunteer program connected to primary care; and (c) explore the outcomes and value of volunteering with Health TAPESTRY. This program had volunteers working with older adults.	Leaders; volunteer health connectors	At least 18 years old
	Gaber et al. (32)	Qualitative Research: Level 2: Group Qualitative Studies with more rigor- prolonged engagement with participants and triangulation of data (multiple sources)	Not reported	Health TAPESTRY is a community-based intervention rooted in primary care which aims to help people stay healthier for longer in the places where they live This program had volunteers working with older adults.	Health connectors- these community volunteers are considered extensions of the primary care team.	Participants were recruited from the pool of Health TAPESTRY volunteers who were at least 18 years old (or undergraduate students who were under the age of 18).
Project HEAL	Holt et al. (33)	Descriptive Research: Level 2: Association, Correlation studies	1—pilot	To describe the process of translating the series of three cancer communication interventions into one coherent, branded strategy for training peer CHAs with two delivery mechanisms. This program had volunteers working with cancer survivors.	CHAs	Not described for CHAs
	Santos et al. (34)	Experimental Research Level 2: Individual (blinded) Randomized Control Trials	2012–2014	To describe the feasibility of a Web-based portal for training peer CHAs. This program had volunteers working with cancer survivors.	CHAs	Self-identified as African American; older than 21 years; regularly attended the enrolled church; able to complete Project HEAL training; had regular access to the Internet and felt comfortable completing Web-based training activities
	Santos et al. (35)	Experimental Research: Level 2: Individuals (blinded) Randomized Control Trial	Not reported	Report on adoption, reach, and implementation outcomes at the organizational and participant levels from an implementation trial in which lay peer CHAs were trained using traditional classroom didactic methods compared with a new web-based system. This program had volunteers working with cancer survivors.	CHAs	African American; between 40 and 75; no personal history of breast, prostate, or colorectal cancer; able to attend 3-workshop series; able to complete project surveys
	Holt et al. (36)	Experimental Research: Level 2: Individuals (blinded) Randomized Control Trial	2014–2016	The current project used the Internet to train and certify CHAs and aimed to determine the relative efficacy of a new technology-based training as compared with the traditional in-person approach. This program had volunteers working with cancer survivors.	CHAs	CHA eligibility criteria included being at least 21 years old, a member of the church, regular access to the Internet and able to complete web-based training activities, and self-identifying as African American.
Connect2	Abeypala et al. (37)	Outcome Research: Level 4: One group pre-post studies	Not reported	Connect 2 was developed as a pilot telephone peer support service for people with type 2 diabetes. It aims to alleviate anxiety around diagnosis for newly diagnosed patients and improve glycemic control and adherence to diabetes management practices. This program had volunteers working with people with diabetes.	Peer supporter	Diagnosed with type 2 diabetes and adhering to standard management practices
Online diabetes peer education program	Bachmann (38)	Outcome Research: Level 4: One group pre-post studies	2006–2009	The purpose of this study will be to examine the feasibility of online training for community diabetes/peer educators—using the National Diabetes Education Program curriculum via a pilot with diabetics and an evaluation of the program by diabetics and professionals. This program had volunteers working with people with diabetes.	Community diabetes/peer educators	African American diabetics age 18 or above, a high school graduate, home computer/Internet access, able to commit to participating in an intensive diabetes self-management peer education training program
HOPE	Jaganath el al. (39)	Descriptive Research: Level 4: Individual Case Studies	Not reported	To train peer leaders in the HOPE study, a new curriculum was created that focused on how to utilize social media websites for outreach to at-risk populations. We describe here the components of the curriculum and the method of evaluation. This program had volunteers working with people with HIV/AIDS.	Peer leader	(1) Male (2) Over 18 years old (3) African-American or Latino (4) Has had sex with a man in the last 12 months (5) Lives in Los Angeles area (6) Experience using Facebook (7) Experience as a Community- Popular Opinion Leader or ability to be a leader in their community (8) Interest in using social networking to educate others about health
P4 for Women	Renfro et al. (40)	Outcome Research: Level 4: One group pre-post studies	February 2015-July 2016	Digitize a traditional face-to-face Training of Facilitators of a faith-based HIV prevention program for young African American women to a digital platform and to describe the MEDIA model, which guided the digitization process This program had volunteers working with people with HIV/AIDS.	Facilitator, Health Educator	Churches must (a) have a predominantly African American congregation according to current membership records; (b) be located in the five Atlanta metropolitan counties with the highest HIV prevalence rates; (c) provide pastoral consent; and (d) agree to the 1-year research participation period.
Women's health information center	McGraw and Jenks-Brown (41)	Descriptive Research: Level 4: Individual Case Studies	2004	Describes the creation of an online tutorial for volunteers who serve the patrons of consumer health information centers. This program had volunteers working women.	Consumer Health Information Center Volunteers	Female university students and women from the community
Foundations of violence against women	Etherington et al. (42)	Qualitative Research Level 2A: Group Qualitative Studies with more rigor: Prolonged engagement with participants	2016	This pilot study examines the efficacy of an online VAW training course using a comparison group design to determine improvements in knowledge, attitudes, and application of learning. This program had volunteers working women.	Anyone that works with women who experience violence	individuals working or planning to work with survivors of Intimate Partner Violence including students, volunteers, and professionals.
Infotility	Grunberg et al. (43)	Descriptive Research; Level 2: Association, Correlational studies	Not reported	To outline the development and evaluation of an online infertility peer supporter training program. This program had volunteers working with people experiencing infertility.	Peer supporter	(1) personal history of infertility, (2) ability to read and write in English and/or French, (3) ability to volunteer 4 h per week, and (4) access to Internet technology for app use (e.g., smartphone, laptop).
Walk with Ease	Conte et al. (44)	Outcome Research: Level 4: One group pre-post studies	2012–2015	To explore the effect of recruitment strategies, training methods, and volunteer characteristics on WWE initiation and describe experiences of volunteers who led WWE. This program had volunteers working with people with Arthritis.	Lay leader	Volunteers had to obtain leader certification, CPR certification, and sign a commitment to lead or co-lead at least one WWE course within 1 year of their training. There were no other prerequisites.
STAR e-learning	Hattink et al. (45)	Experimental Research: Level 2: Individual (blinded) Randomized Controlled Trials	2010–2014	Evaluate the user friendliness, usefulness, and impact of STAR with informal caregivers, volunteers, and professional caregivers. This program had volunteers working with people with Dementia.	Volunteers in dementia care	(1) were sufficiently computer literate to utilize the STAR website and (2) were currently an informal caregiver for someone with dementia living in the community, or a volunteer working with people with dementia with direct contact with community-dwelling people with dementia, or a professional caregiver for people with dementia with direct contact with community-dwelling people with dementia.
Veterans Health Administration's DEMPS	Schmitz et al. (46)	Outcome Research: Level 4: One group pre-post studies	2012	To (1) better understand DEMPS volunteers' training mode utilization and attitudes about training, and (2) identify areas of improvement for DEMPS' training program. This program had volunteers working with people with experiencing disaster.	DEMPS Volunteers	

*Health-TAPESTRY, Health Teams Advancing Patient Experience: Strengthening Quality HEAL- Health through Early Awareness and Learning; CHA, Community Health Advisors; HOPE, Harnessing Online Peer Education; WHICH, Women's Health Information Center; VAW, Violence Against Women; WWE, Walk with Ease; STAR, Skills Training and Reskilling; DEMPS, Disaster Emergency Medical Personnel System*.

Tables 1, 2 present information from articles found in the database search; Table 1 presents information on the basics of each identified program, which includes populations targeted by the program, aims of project/study, role of volunteer etc. Table 2 presents information on the online training for these programs, including details of training, materials used, and topics covered as well as acceptability promoting factors and barriers to feasibility.

**Table 2:** Characteristics of online volunteer training delivery from database search.

**References**	**Details of Training: Length/Frequency/Delivery mode**	**Topics covered**	**Materials used**	**Acceptability promoting factors**	**Barriers to feasibility**
Oliver et al. (30)	Initial Training followed by ongoing learning modules 11.5 h of training Mixture of online and in-person training	Forming supportive relationships with clients, fathering information about client's health needs and health-related goals, assist client with set-up of their own personal health record, share information about community resources, provide motivational support	Live action scenarios, PowerPoint presentation with voice over and testimonies from past volunteers	Quality of Interaction, ease of use/simplicity	Not reported
Dolovich et al. (31)	Volunteers took between 91 and 466 min to complete all modules Ongoing learning sessions were held based on volunteer interest and need. Online modules were self-paced	Effective communication, Intercultural communication, conflict resolution, privacy and confidentiality, health and safety, using the HL-App and other eHealth technologies, mental health, health promotion, disease prevention and risk factors, diabetes, hypertension, general health factors, motivational interviewing techniques.	The online Volunteer Learning Center (VLC) included 14 video and quiz modules; Training manual.	Feeling effective	Not reported
Gaber et al. (32)	A multi- modal training program which included: a 2-h interactive, in-person training session followed by online learning through the Health TAPESTRY Virtual Learning Center (VLC) which included video modules and quizzes. Time it took to complete self-paced modules was not recorded.	Examples of content for training included communications, program implementation and tools, health and safety, technology use, and privacy and confidentiality; for further detail	A 2-h inter- active, in-person training session; online learning through the Health TAPESTRY Virtual Learning Center (VLC) including video modules and quizzes; and a paper-based manual.	Not reported	Not reported
Holt et al. (33)	The web-based training is self-paced through PowerPoint slides, which was done self-paced Knowledge examination to be Community Health Advisor certified (multiple choice exam on risk factors, symptoms, screening methods)	Introduction to project HEAL, overview of cancer, breast cancer, prostate cancer, colorectal cancer, health beliefs, spirituality and health, adult education, leadership skills, communication skills, conducting the workshop, documentation, and ethical issues.	Web-based participants receive username and password for training portal, as well as a hard copy of Community Health Advisor Training Manual, Health Ministry Guide, and Cancer Resource Guide to complement their online training process.	Not reported	Not reported
Santos et al. (34)	Training took on average 26 days, which included 13 training videos. This was done self-paced.	Overview of cancer, breast cancer, leadership skills, ethical issues	Intervention materials (e.g., cancer resource guide outlining local health care resources) and sets of PowerPoint slides for 3 workshops (i.e., cancer overview, breast and prostate cancer, and colorectal cancer) are provided in a downloadable format	Not reported	Not reported
Santos et al. (35)	Two × 3 h training or 1 × 6 h training which was self-paced	Overview of cancer, breast/prostate/colorectal cancer/how to conduct a workshop etc.	13 modules (delivered in web-based training videos in Technology condition, and delivered in classroom in Traditional condition).	Ease of use/simplicity	Not reported
Holt et al. (36)	6+ h Knowledge examination following training It was not recorded it if was self- paced or live	Overview of cancer, specific cancer information (three most common types), conducting workshop, communication, ethics, leadership	Narrated PowerPoint slides	Not reported	Not reported
Abeypala et al. (37)	Volunteers were assessed before worked with actual participants Length of training and delivery was not recorded	Training consisted of information about type 2 diabetes management and motivational interviewing skills.	Not reported	Not reported	Not reported
Bachmann (38)	6 weeks, twice-a-week, 12 sessions total, all sessions were self- paced	Power to prevent; supermarket; exercise/walking; restaurants; cooking at home; types and ABC; symptoms, prevention and physical activity; physical activity for families; portion sizes/cooking from home—a review; partnering with your provider	A home computer/Internet access, dedicated email accounts for study, 13 training videos, pre- and post-lesson quizzes, pre- and post-training measures	Ease of use/simplicity	Technology challenges/complex training schedule
Jaganath el al. (39)	Training lasted 12 weeks Self-paced and live (education modules, interactive discussion, modeling, role play, repetition)	Knowledge: HIV/AIDS prevalence in the MSM community, methods of transmission, testing, treatment, and prevention strategies, and myths around HIV/AIDS; Social context—basic epidemiology facing Latino and African-American MSM, the unique cultural obstacles, and the consequences of stigma; Communication: How to initiate a conversation, online etiquette	Role playing and educational modules	Ease of use/simplicity, quality of interaction	Not reported
Renfro et al. (40)	4-h video training, divided into shorter segments, or video chapters. Took 4 days (36 h of effort)	Chapter 1: Welcome and Course Introduction, Chapter 2: Preparing to Implement, Chapter 3: Facilitation Skills, Chapter 4: Using the Training Materials (+ Appendices)	Supplemental materials included a written user guide, a website, and a facilitation toolkit.	Not reported	Not reported
McGraw and Jenks-Brown (41)	Training is self-paced Length and frequency of training was not reported	Seven lessons: about WHICH, office orientation, ethics, answering questions, WHICH resources, evaluating online health information, web resources.	Print manual	Ease of use/simplicity	Computer skills
Etherington et al. (42)	4-weeks (typically spend 2.5 h per week on course) Training is self-paced	Course topics include intersectional feminism and Violence Against Women, portfolio development, ethical decision making, record-keeping, self-reflective practice, feminist analysis of mental health issues, harm reduction, and risk assessment and safety planning	Online learning portal content		
Grunberg et al. (43)	Training was done one time for 4 h Training was a mixture of self-paced and live	Peer supporter role; factual information about infertility; the use of different types of peer support (e.g., emotional support; expected conduct (e.g., rules, guidelines, ways to monitor the forum for 'red flags'); procedures for contacting peer support coordinators; FAQ; glossary of medical terms and abbreviations.	Training manual, training webinar, practice discussion posts The manual included example posts and acceptable responses to 10 fertility related topics: medical; lifestyle; mental health; couple's issues; sex, intimacy, and infertility; work; talking to friends and family; culture and infertility; pregnancy loss; and, the fertility journey.	Quality of Interaction	Technology challenges
Conte et al. (44)	Training is self-paced 10-question multiple choice test to get certification	Proper stretching techniques, basic information about arthritis and physical activity	Narrated slides and videos, training manual	Not reported	Computer skills, technology challenges
Hattink et al. (45)	8 online modules, self-paced	(1) What is dementia? (2) Living with dementia, (3) Getting a diagnosis and why it is important, (4) Practical difficulties in daily life and how to help by best practice, (5) The emotional impact of dementia: how adaptation and coping influences behavior and mood (6) Support strategies to help people cope with consequences of dementia, (7) Positive and empathic communication, (8) Emotional impact and looking after yourself	STAR Training Portal		Computer skills, technology challenges
Schmitz et al. (46)	Training was self-paced; field exercises and classroom lecture are live	Not reported	Not reported	Not reported	Not reported

*Health-TAPESTRY Health Teams Advancing Patient Experience: Strengthening Quality HEAL, Health through Early Awareness and Learning; CHA, Community Health Advisors; HOPE, Harnessing Online Peer Education; WHICH, Women's Health Information Center; VAW, Violence Against Women; WWE, Walk with Ease; STAR, Skills Training and Reskilling; DEMPS, Disaster Emergency Medical Personnel System*.

The following components of the training programs have been summarized below. For the purposes of this review, the literature search process was broken down into two separate search strategies that have been outlined in the methods section above: (A) Database search strategy and (B) Online training Program search strategy.

A summary of the extracted data from the included articles identified in the database search strategy is provided below, more detailed information is in Table 2. This summary is followed by an evaluation of the feasibility and acceptability of the programs described by the articles, this is organized by the themes: (1) feasibility-promoting factors; (2) barriers to feasibility; (3) acceptability-promoting factors; and (4) barriers to acceptability.

A summary of the online training programs search results is provided next. In addition, there is detailed information in the data extraction table (Table 3). The summary is followed by an assessment of feasibility and acceptability in the online training programs identified (for detailed information see Table 4).

**Table 3:** Characteristics of existing online volunteer training programs.

**Organization**	**Program name**	**Aim/Purpose**	**Volunteer role/Criteria for volunteers**	**Inclusion criteria for volunteers**	**Years delivered**
Calgary Seniors Resource Society	Seniors connect- caregivers	To keep seniors healthy, independent and safe in their homes as long as possible	Volunteer “connectors” ensure seniors are connected with people who care on a regular basis and receive the support they need when they need it	Over the age of 18, commit to the program for a minimum of 6 months	Organization has been in operation for “over 20 years”
Canadian Hospice Palliative Care Association	Hospice palliative care volunteers: A training program—online version	CHPCA is the national leader in the pursuit of quality hospice palliative care in Canada through: public policy, education, knowledge translation, awareness, and collaboration.	A Training Program has been developed to help hospice palliative care programs across the country ensure that volunteers receive the consistent training and information they need to provide high quality services	Not reported	Manual is dated 2012 (2012–Present)
Circle of Care	Friendly visitors	Dedicated to supporting independence at home and enhancing quality of life for people in the community	Friendly visitor—visiting him or her in their own home. Clients and volunteers are matched with someone of similar interests and location is prioritized based on your requirements	Not reported	Not reported
Red Cross Alliance	Health TAPESTRY	Health TAPESTRY works to help people stay healthier for longer in the places where they live. The health goals of our clients are the basis for engaging people in meaningful ways. We are doing this through an adaptable model of health care that connects people, their health care team, volunteers and their community, through the support of technology.	Visiting clients where they live, along with a fellow volunteer; gathering information using a tablet and Health TAPESTRY technology, like the TAP-App; helping clients set-up a personal health record (PHR) with the online kindred PHR; helping motivate clients to reach their health goals; connecting clients to community resources.	Be at least 18 years of age	2017- Present
Hospice Toronto	Hospice Toronto	Enables access to appropriate care and support for those with serious illness and their caregivers when they need it, where they need it, and whoever they are. We achieve this through our culture of caring and discovery that is fueled by passionate, dedicated volunteers, imaginative partnerships, and a focus on research and innovation.	Various: complementary therapy volunteers such as massage therapists or Reiki, Expressive arts therapist; or home help volunteers and creating caring communities helpers	Over the age of 18, and interviewed to be deemed a “good fit” for the volunteer program	2020- Present (Training became online for COVID)
YeeHong Center for Geriatric Care	Friendly visitors	To enable Chinese Canadian and other seniors to live their lives to the fullest, healthy, independent and dignified, through our continuum of excellent, culturally appropriate care.	Visit and chat with residents in the nursing home or isolated seniors in the community.	Be over the age of 18, have a negative vulnerable sector check and TB test	10+ years according to 2020 version of volunteer handbook.

**Table 4:** Training delivery characteristics of existing online volunteer training programs.

**Organization/Study Title**	**Program name**	**Details Of Training Frequency/Length/Delivery mode**	**Topic covered**	**Materials**	**Feasibility promoting factors**
Calgary Seniors Resource Society	Seniors Connect- Caregivers	12-min online video Numerous videos to go back to Self-paced	Seniors Connected—Caregiving video: Roles of a caregiver; caregiver significance; caregiver burnout; understanding the challenges; warning signs and risks	Online videos	Topic relevancy, low attrition, and high engagement
Canadian Hospice Palliative Care Association	Hospice palliative care volunteers: A training program—online version	Nine learning modules Accompanying toolkit (to be done on your own after training) Self-guided, either with print material or online	Goals of hospice palliative care; volunteer role and boundaries; effective communication; privacy and confidentiality; how attitudes toward death and dying can shape their support of the dying person; cultural and spiritual beliefs in death and dying; how to provide spiritual care but also know when to ask for help; managing pain and symptoms; infection control; the context of being in someone's home; loss of appetite; transferring and lifting the person; stages of grief; helping children and older people cope with grief; stress management	Hospice Palliative Care Volunteers: A Training Program (printed and online copies available); contains a series of nine learning modules specific to the Volunteer's role in Hospice Palliative Care, and an accompanying toolkit that can be used to guide the Volunteer through the learning modules.	Topic relevancy
Circle of Care	Friendly Visitors	One time training ~30 min Self-paced	General role responsibilities	Printable copy of role guidelines/expectations	Not reported
Red Cross Alliance	Health TAPESTRY	Combination of in-person and online modules. Continued access to online information may be offered to volunteers on new topics or areas of need. Volunteer passes quizzes to be approved to work with clients	Privacy and confidentiality, effective communication, conflict resolution, data gathering tools, motivational interviewing, boundaries, risk management and health and safety, mental health, TAPESTRY technology	There are also Additional Resources to learn more about a topic and Interactive Community Pages with discussion boards and the chance to connect with other volunteers and staff.	Topic Relevance
Hospice Toronto	Hospice Toronto	One-time training followed by on-going support from community development coordinator, varies by role. The complementary therapy volunteers undergo 35+ h of “core hospice training” and the HHV/CCCHs do 14+ h of training Training was completed live with additional readings.	Introduction to hospice, palliative care, role of the volunteer, understanding boundaries, communication skills, pain and symptom management, understanding the dying process, spirituality, grief and bereavement, care for the caregiver, family dynamics, ethics, impact Illness and psychosocial concerns, cultural considerations, infection prevention and control, body mechanics, assists and hands on care	A magnitude of resources available online	Topic relevance
YeeHong Centre for Geriatric Care	Friendly Visitors	There is online volunteer training that takes ~30 min to complete. There are ongoing training sessions throughout the year to enhance volunteers' skills in delivering service. Some training sessions are mandatory. Orientation is live but learning modules are self-paced	Residents' bill of rights; privacy and confidentiality, accessible customer service, abuse and zero tolerance, mandatory reporting and whistle blowing, responsive behaviors, universal infection control practice, client and workplace safety, emergency protocol, incident reporting, complaint procedure	Handout (PDF), volunteer handbook, virtual presentation (Prezi)	Not reported

### Summary of Included Articles

#### Volunteer Roles and Responsibilities

Volunteer trainees filled many specific roles such as Peer Supporter (33, 37, 42), Peer Educator (47), Lay/Peer Leader (31, 40, 44), Volunteer Health Connector (31), Community Health Advisor (33, 35, 36), Consumer Health Information Center Volunteer (41), and Disaster Emergency Medical Personnel System Volunteer (46). Responsibilities included motivational interviewing over the telephone (37), visiting clients at home (30–32), or providing support to clients virtually (39, 43), all with the objective of contributing to the delivery of a health-promoting program [i.e., supportive environments that allow people and communities to adopt healthy behaviors; (48)] to adults in the community.

#### Delivery of Training

Due to our inclusion criteria, there was no exclusively live training for these roles. However, the online training was sometimes combined with simultaneous in-person (33, 36, 42) or hybrid (46) training to assess differences in implementation from delivery method. Training delivery was delivered equally between a multi-medium platform (e.g., live components and self-paced components) that enabled flexibility for training (34, 35, 38, 42, 46), and solely self-paced (31, 33, 36, 44, 45). When training was self-paced, materials included educational videos, quizzes, narrated PowerPoint slides, training manual; practice sessions with role playing; interactive discussions; and recorded webinars.

#### Evaluation of Feasibility and Acceptability

Nine peer-reviewed articles evaluated the implementation of their training program. The findings indicated that online training was largely feasible (*N* = 7/9). Only two studies reported that online training for their program was not feasible (38, 44), primarily due to recruitment and attrition of volunteers. Conte (44) reported feasibility was affected by a lack of volunteer sustainability resulting in poor uptake. Bachmann (38) concluded that the current structure and format of the training program was not feasible, due to problems with volunteer recruitment, a low completion rate (8%), and issues with volunteers' completion of every component of the intervention. Eight studies did not evaluate or explicitly report on feasibility. Acceptability was evaluated less frequently (*N* = 6) than feasibility, although all studies that evaluated acceptability reported positive results (30–32, 34, 43, 44). A majority of these studies were for the same training program (31, 33–36, 49).

Feasibility was assessed with process measures (31, 34), volunteer reflections (31), volunteers' understanding of their roles and responsibilities (31), client perspectives on the volunteer program (31), and cost effectiveness (30, 41, 43). Acceptability was measured through volunteer perceptions of ease of use (34, 44), ease of understanding (34, 44), acceptability of the workload or commitment (31, 34), and perceptions of effectiveness in volunteer role (30).

#### Thematic Analysis

Information on accessibility and feasibility was organized into four themes (1) Feasibility Promoting Factors; (2) Barriers to Feasibility; (3) Acceptability Promoting Factors; and (4) Barriers to Acceptability.

##### Feasibility Promoting Factors

Feasibility promoting factors were defined as factors that enable success of a program through compatibility with available resources (21–25) and included topic relevancy, preventing attrition/promoting engagement of volunteers, and a leadership/mentorship component.

###### Topic Relevancy.

Topics were considered more relevant when program leads were consulted to aid in identifying online strategies and tools that fit their program (40). When content was designed based on trainee interest or gap in knowledge it helped maintain interest (30). Volunteers undergoing training were appreciative of relatable content that reflected situations they expected to find themselves in when applying their training (38). Volunteers liked the opportunity to contribute to future training designs by giving feedback (32, 41), which helped with successful training (39).

###### Preventing Attrition/Promoting Engagement.

Instructor engagement, such as enthusiasm and endorsement of the overall program or program content helped volunteers stay motivated and commit to the workload (38). Diversity in training methods (e.g., online, role play, in-person, written manual) was appreciated by volunteer trainees for maintaining interest (31). In one intervention, TAPESTRY (31, 49), previous experience with online training (such as being in the second year of program implementation) increased odds of successful training completion (44). Having an interactive, participatory component [e.g., interaction or role play with others or having discussion board; (30, 32, 39, 43)] helped volunteers remain engaged.

###### Leadership/Mentorship Component.

A mentorship or leadership component [e.g., a champion of the program or an experienced volunteer mentoring an incoming volunteer; (31, 44)] promoted volunteers' participation in training.

##### Barriers to Feasibility

A common barrier to feasibility was perceived lack of consideration for varying levels of technical literacy or computer skills (41, 44, 45). For instance, technical difficulties with the online platform meant that videos or other content loaded slowly and that the structure of the online program was overly complex (38, 43–45). The in-person design may not have been adapted sufficiently for online delivery (38).

##### Acceptability Promoting Factors

Acceptability promoting factors enable benefits that are identifiable and/or address systemic, social, or educational challenges. Acceptability promoting factors included ease of use/simplicity, quality of interaction and volunteers perception of effectiveness (see Table 2).

###### Ease of Use/Simplicity.

The online platform was simple and easy to navigate (41), and was accessible on different platforms [e.g., iOs, Android, computer; (35)]. A comfortable pace of teaching helped facilitate learning (38, 39). Using multiple mediums to convey information during training and using short, digestible videos were helpful to volunteers (38).

###### Quality of Interaction.

Volunteers liked the use of social media for interaction between volunteers, such as role play (39) with client simulation scenarios (32). Involvement in online discussion with clients was a welcome interaction (43). Volunteer trainees were paired with an experienced volunteer (32), such as a “clinician champion” (30).

###### Volunteers Perception of Effectiveness.

Volunteers in the TAPESTRY program (31) understood themselves as healthcare system connectors, feeling fulfilled with their contributions and learning new skills. They recognized their own utility and felt their involvement was positive and reported that volunteering improved their own health (31). The TAPESTRY volunteers also gained knowledge, self-efficacy and skills from their training, which received largely positive evaluation (31).

##### Barriers to Acceptability

Some volunteers thought the demand of the volunteer role was too high for an unpaid role given their health education background (44) and that too much time elapsed between training and application of training (32). Some volunteers felt clients had complex needs that they could not address sufficiently and that not all clients or recipients were amenable to volunteer support (31), indicating that some perception of insufficient training exists among volunteers trained online. Conte et al. (44) also determined that those without a health education background typically sought out more resources than what was given to them.

### Method B: Results of Existing Online Training Programs

#### Summary

The integrative review was complemented by a search of existing online training programs in Canada. The details on inclusion/ exclusion of programs are shown in Figure 1. Programs were excluded when they were a hub for other satellite programs that offered the training, training was actually offered in-person, or the program was not for volunteers. Tables 3, 4 present information from articles found in the existing online training programs search. Table 3 specifically highlights the aim of the programs and Table 4 presents details of online training delivery for each program.

Given the six online training programs, all but one used video(s) as a means to convey educational content to the volunteers. The training video(s) were typically one single video (50–52), some within a one-module training sessions (50, 51) or as part of a modular training design (52), while two programs offered a series of short videos (53, 54). On-going training and support were offered in two programs (52, 54) beyond the initial training session. All program training was designed for self-paced learning except for one (54), which, depending on the specialization (e.g., therapist volunteer vs. general volunteer), could include up to 35 h of training with some live group sessions. Due to the self-paced nature, most programs offered resources to enhance learning, such as a volunteer handbook (52, 55) or the module information in printable format (51, 52). One program (53) quizzed trainees following the educational videos and offered online discussion boards for volunteer interaction.

### Integrating Findings on Program Acceptability and Feasibility From the Peer-Reviewed Literature to Existing Online Volunteer Training Programs

Based on the literature (see Table 4), we know that feasibility-promoting factors are topic relevancy (30, 32, 39–41), volunteer engagement/low attrition (31, 38, 49), and inclusion of a leadership/mentorship component (31, 44). The primary barrier to feasibility was with technology, specifically, having varying levels of computer skills across volunteers or having issues with delivering training components such as uploading videos (38, 41, 43–45). Acceptability-promoting factors are ease of use (35, 38, 39, 41), interaction quality (30, 32, 39, 43), and *feeling effective* (31). Barriers to acceptability were feelings of: (a) the volunteer workload being too high for an unpaid role (44), (b) too much time elapsing between training and application of the training (32), and (c) that clients had complex needs that volunteer support could not sufficiently address (31).

Based on our review of the six pre-existing online programs, none showed all three feasibility promoting factors identified from the literature (i.e., topic relevancy, volunteer low attrition/high engagement, leadership/mentorship component) and five showed some barriers to feasibility identified from the literature (i.e., technology challenges/complex training schedule, computer skills). Two programs (Circle of Care; Yeehong Center for Geriatric Care) lacked the information necessary to assess the presence of feasibility promoting factors and seven programs lacked the information necessary to assess the presence of acceptability promoting factors. Two programs (Senior Connect; Health TAPESTRY) showed two factors that promote feasibility; both programs included relevant, varied content, one included a commitment requirement (preventing attrition), and one included a volunteer-peer (i.e., a fellow volunteer who progressed through training as a peer learner). Four programs reported technology challenges that impeded acceptability (38, 43–45).

## Discussion

The aim of this review was to examine the feasibility and acceptability of community-based organizations adopting an online training format for their volunteers by synthesizing evidence from the research and gray literature on online training/coaching/mentoring programs for volunteers delivering services to adults. In particular, we wanted to identify facilitators and barriers to feasible and acceptable online training for volunteers who support older adults in the community.

From the literature, we learned that online volunteer training programs factors promoting feasibility and acceptability were (a) topic relevancy of training (31, 38, 49); (b) an interactive, participatory component to training [e.g., interaction or role play with others, having discussion board; (30, 32, 39, 43)]; and (c)mentorship or leadership [e.g., having a champion of the program, or having an experienced volunteer mentor incoming volunteers; (31, 44)]. Major challenges to successful transition to online training included: (a) poor technology and software performance (38, 41, 43–45) or that was difficult to learn and use (35, 38, 39, 41); (b) poor or no interaction (30, 32, 39, 43); and (c) perceptions of ineffectiveness or overwhelming workload (31).

The online programs reviewed showed little adherence to the evidence from our database search of training methods for online delivery. For example, feasibility is promoted when the online training structure requires high engagement from the volunteers (31, 38, 49), but programs' educational materials were typically a single video (50–52) within a one-module training session (50, 51) or as part of a multi-module training design (52). Further, on-going training and support were offered in only two programs (52, 54) beyond the initial training session, all program training was designed for self-paced learning except for one program (54), and only one program (53) assessed volunteers' knowledge of the material or offered online discussion boards for volunteer interaction.

Acceptability may be enhanced by improving interaction quality (30, 32, 39, 43) and volunteers feeling effective in their support role (31). Few online programs implemented sustained teaching methods (e.g., follow-up training or support, online discussion boards). In fact, a barrier to acceptability was volunteers feeling clients had complex needs which volunteer support could not sufficiently address (31). Systematic support and follow-up training would be beneficial for promoting volunteers' self-efficacy, as feasibility is promoted when volunteers feel effective in their supportive role.

Although topic relevancy of the training was identified as a major contributor to feasibility in our database search (30, 32, 39–41), only two programs offered thematic or topic-specific videos (53, 54). Additional identified technological barriers to feasibility were addressing the varying levels of computer skills across volunteers or having issues with delivering training components such as uploading videos (38, 41, 43–45). Most programs attempted to address this by offering resources that enhanced the online training, such as providing a volunteer handbook (52, 55) and educational materials in printable format (51, 52). For example, supplemental materials such as a volunteer handbook or educational materials can provide additional support to volunteers who have low computer skills.

None of the online programs reviewed exhibited all three feasibility promoting factors identified from the literature (i.e., topic relevancy, low attrition and high engagement, leadership component), although two programs (51, 52) lacked the information necessary to assess them.

### Recommendations for Increasing Feasibility and Acceptability Promoting Factors of Online Delivery of Volunteer Training

There is evidence that feasibility is promoted when topic relevancy (30, 32, 39–41) increases volunteer engagement and participation (31, 38, 49) and includes a leadership/mentorship component (31, 44). Additionally, there is evidence that online training acceptability is promoted when the technology and software (38, 41, 43–45) are easy to use (35, 38, 39, 41), enables quality interactions (30, 32, 39, 43), and perceptions of effectiveness (31) are obtained through testing volunteers' knowledge gained following training (e.g., module quizzes).

### Limitations and Future Directions

This review was limited by the search strategy. Our search strategy was developed to identify existing online training programs that were applicable to older persons and peer-reviewed evidence on online training programs applicable to all adults, thus our conclusion is based on evidence garnered from a two slightly different demographic profiles. As our inclusion criteria selected only English-written articles, the evidence synthesized in this review is limited to English speaking regions. None of the online programs reviewed showed evidence of any evaluation of acceptability and feasibility, suggesting that there has been no formal evaluation done on these programs. Thus, our ability to identify feasibility and acceptability promoting or impeding factors in these programs was limited to our interpretations of the available materials. All of the articles reviewed in the database search were based on the researchers' perspectives, resulting in a lack of perspectives from volunteer trainees themselves. This limited our assessment of acceptability, as acceptability is optimally defined and measured by end-users' experience with the training program. A majority of the articles from the peer-reviewed literature were for the same training program. This may skew our findings toward those programs, in that counting programs more than once could bias the identified themes toward those programs and minimize the impact of the remaining programs. For example, a program that is counted more than once may exhibit specific feasibility and acceptability factors that are not present in another program, but are counted as being “frequently reported” due to the program being counted more than once. This bias is due to the lack of published evaluations of online training programs. To reduce this bias there needs to be more evaluation research on volunteer training programs. Programs need to assess and report their evaluations of feasibility and acceptability to enable appropriate design and implementation of online training programs. It would also be beneficial for researchers to publish the program protocol and evaluation. This would facilitate a more thorough assessment of feasibility and acceptability that begins with program design and planning. Finally a synthesis of online training for volunteers providing supports to other populations would improve generalizability.

## Conclusion

Our synthesis supports the conclusion that online volunteer training is feasible and acceptable for community-based organizations that support adults, particularly if facilitators of feasibility are present. There is evidence that feasibility is facilitated when topic relevancy promotes engagement/low attrition and includes a leadership/mentorship component. Additionally, there is evidence that acceptability is facilitated when there is sound technology and software that is easy to use and enables interaction quality and perceptions of effectiveness. Based on our review of the pre-existing online programs, none of the programs showed all three feasibility promoting factors identified from the literature. Although it may be feasible and acceptable for community-based organizations to adopt an online training format for their volunteers, the online programs reviewed showed little adherence to the evidence from our database search of training methods for online delivery.

## Author Contributions

BP, WD, and GW conceived the study and secured its funding. TH, JL, EK, and GW screened articles and analyzed data. TH, JL, EK, BP, WD, and GW contributed to drafting and revising the manuscript. All authors read and approved the final manuscript.

## Funding

This study was funded by the Canadian Institutes of Health Research [#148655] and the Canadian Cancer Research Institute [#704887].

## Conflict of Interest

The authors declare that the research was conducted in the absence of any commercial or financial relationships that could be construed as a potential conflict of interest.

## Publisher's Note

All claims expressed in this article are solely those of the authors and do not necessarily represent those of their affiliated organizations, or those of the publisher, the editors and the reviewers. Any product that may be evaluated in this article, or claim that may be made by its manufacturer, is not guaranteed or endorsed by the publisher.

## Appendix

**Table A1:** Data extraction template.

**Study ID**	**Type of** **study or article**	**Aim of study or program**	**Type of program**	**Name of program**	**Content or subject matter of training program**	**Materials** **used in** **training program**	**Any follow-ups or additions to training**	**Type of volunteers taking training**	**How does the volunteer deliver program after training**	**What outcomes are assessed?**
